# Indentation Induced Mechanical Behavior of Spark Plasma Sintered WC-Co Cemented Carbides Alloyed with Cr_3_C_2_, TaC-NbC, TiC, and VC

**DOI:** 10.3390/ma14010217

**Published:** 2021-01-05

**Authors:** Piotr Siwak

**Affiliations:** Faculty of Mechanical Engineering, Poznan University of Technology, 5 Marii Sklodowskiej-Curie Square, 60-965 Poznan, Poland; piotr.siwak@put.poznan.pl; Tel.: +48-61-665-22-61

**Keywords:** mechanical behavior, hardness, indentation size effect, cemented carbides, spark plasma sintering

## Abstract

The focus of this paper is on examining the mechanical behavior of spark plasma sintered WC-Co based composites doped with Cr_3_C_2_, TaC-NbC, TiC, and VC, as well as defining some parameters characterizing deformation and fracture processes during hardness measurement. The calculated microhardness of WC-Co cemented carbides for all the studied compositions is found to be higher than the results obtained during hardness testing. Therefore, the ratio of the experimental and calculated values of microhardness is shown to be an approximate indication of WC-Co cemented carbide sensitivity to damage processes during indentation. Some parameters characterizing the microstructure–microhardness relationship are defined, and the nanomechanical properties of WC-Co cemented carbide phases are examined in order to separate the deformation and fracture processes during the indentation process. Strain gradient linear function parameters are calculated for 10-cycle nanoindentation. It was found that the nanoindentation curve after 10 cycles shows anomalous behavior of the WC grains, which indicates their fracture processes.

## 1. Introduction

The unique combination of high mechanical properties (hardness, compression strength), and wear resistance of WC-Co cemented carbides results in their widespread application in cutting tool materials. The properties of WC-Co cemented carbides depend on the powder metallurgy technology (milling, mixing, compaction, sintering), cermet composition (first of all the Co content), WC and Co grain size, as well as additives which can be varied to improve the microstructure and increase the cermet properties.

Most of the applications of WC-Co composites used for various machining, drilling, mining, forging and stamping tools require high hardness. Moreover, the elastic, plastic deformation and indentation forced cracking behavior exhibited by WC-Co composites need to be monitored by hardness and nanohardness examinations [1]. The main goal of the hardness and nanohardness studies is to establish and evaluate the relationship of the microstructure–properties on the basis of the deformation and fracture processes of the WC-Co composite constitutive phases due to high local contact stresses, strains and temperatures, which result in tool surface wear [2]. From this viewpoint, the examination of indentation induced deformation and the fracture mechanisms of each phase (WC grains and the binder phase) is of great importance [3].

### 1.1. Microstructure–Microhardness Relationship

Determining the WC-Co cemented carbide microstructure–hardness relationship allows prediction of the performance of composite materials made of contrasting phases. The combination of WC carbides and a tough, energy absorbing Co binder phase results in obtaining a composite which has some of the best characteristics of each phase. The mechanical behavior of WC-Co cemented carbides during indentation is determined in numerous works reviewed by Engqvist et al. [4], which show that the WC-Co cemented carbide flow stress falls exponentially with the thickness of the Co binder layers. This effect has been ascertained for a few other ceramic materials consisting of hard crystals bonded with a binder phase. Notwithstanding, no attempts have been made to evaluate the validity of the Hall-Petch [5] and other similar relations to determine the dependence of hardness on the microstructure parameters such as the Co binder thickness (*λ_Co_*) of SPSed WC-Co composites. The universal model of the dependence of WC-Co cemented carbide hardness (*H_CC_*) on *λ_Co_* and the mean WC grain size (*d_WC_*) is presented in [4] as the following relationship:

(1) $$H_{cc}=\left(693+\frac{2680}{\sqrt{2.1+d_{WC}}}-H_{Co}\right)e^{-\frac{\lambda_{Co}}{k}}+H_{Co},$$

where *k* is the hardening range factor, *k* = 0.35, and *H_Co_* is the binder hardness. Hence, experimental determination of the microstructure parameters and calculation of the hardness–microstructure function based on Equation (1) is imperative for further analysis.

As shown in paper [6], the high mechanical properties of the WC-Co cemented carbide such as strength toughness and wear resistance are achieved due to its specific microstructure morphology—an interpenetrating network of hard WC crystals and a soft/ductile Co binder phase. Some data on the influence of the microstructural parameters on the WC-Co composite mechanical behavior have been presented, e.g., in [6,7]. For example, based on HR-TEM examination results, the authors of [8] described the possible damage mechanisms of the WC-Co composite constituents during nanoindentation. The main conclusion based on the above results is that the damage process of both the WC carbides and Co binder starts from the first loading and may be recorded at the microscale during hardness measurement. From the current author’s point of view, this damage process will influence the mechanical behavior of the material during indentation loading and will result in a decline in hardness. Unfortunately, detailed information on the loading behavior of cemented carbides is relatively insufficient, in spite of the fact that their sensitivity to damage accumulation during loading process is well described [8,9]. An accumulation of data on WC-Co fatigue behavior is important because the exploitation properties and durability of cemented carbides working in hard loading conditions must be increased. From this viewpoint, examination of the influence of the indentation load on the hardness is believed to shed light on the hardening and damage mechanisms of WC-Co composites.

### 1.2. Indentation Size Effect

Studying the in situ mechanical behavior of WC-Co cemented carbides subjected to wear is a challenging problem due to the limited availability of wear process and microstructure parameter measurement techniques, the complexity of these techniques, in addition to the long experimental period and its high cost [10,11].

As an alternative, worn samples are often used as surrogates to study the microstructure and properties of WC-Co composites [12] at the micro and nanoscale. In an overview [13], Duszová et al. analyze the state-of-the-art results of WC-Co cemented carbide nanohardness measurement and demonstrate the effectiveness of such a nanoscale approach. The indentation size effect (ISE) of hard materials microhardness tests and nanohardness tests of the WC and Co phases is evaluated. Additionally, the effect of the WC crystals orientation on their mechanical behavior is presented. Moreover, the WC and Co phases wear and damage mechanisms as well as their characterization are also analyzed. The unusual mechanical behavior of WC-Co composites is found to be the result of local deformation and crack generation of the WC and Co phases. However, there is still a lack of ISE study at the nanoscale and dependence of the ISE characteristics on the damage parameters of the WC-Co composite microstructure.

ISE characterization is well known to be conducted based on strain gradient plasticity (SGP) theory developed in [14,15]. An SGP model is built on the generation and accumulation of geometrically necessary dislocations (GNDs) due to strain gradients arising during the indentation test. Reducing the indent dimensions leads to an increase in GND density and results in higher hardness values. In accordance with the Nix and Gao (NG) model based on SGP [15], ISE is described as:

(2) $$\frac{H}{H_{0}}=\sqrt{1+\left(\frac{h^{*}}{h}\right)}⊳{\left(\frac{H}{H_{0}}\right)}^{2}=h^{*}\left(\frac{1}{h}\right)+1,$$

where *H*_0_ is the intrinsic hardness determined at the indentation depth *h* → ∞, and h* is the intrinsic length characterizing the strain gradient sensitivity of the material volume beneath the indenter. Two types of dislocations are responsible for the deformation process during indentation: GND and SSD assuming the initial dislocation density is not taken into account. The SSD density is accumulated during the whole deformation process; however, the GND density is high at the beginning of indentation and falls during indentation. For this reason, intrinsic hardness *H_0_* is determined by the density of the SSDs (*ρ_s_*) at a high level of indentation depth. In general, the hardness–equivalent flow stress ratio is well known [16] to be defined by a Tabor coefficient of 3. Therefore, taking into account the classical von Mises plasticity equation, *H*_0_ dependence on the dislocation density may be defined as:

(3) $$\sigma =\sqrt{3}\tau ;H_{o}=3\sigma ⊳H_{o}=3\sqrt{3}\alpha \mu b\sqrt{\left(\rho_{S}\right)},$$

In accordance with Duszová et al. [8], the following deformation and fracture mechanisms may occur during WC-Co composite loading:

1. deformation of the WC phase due to slip line development in the WC grains in different slip systems,
2. fracture of the WC grains by crack nucleation and growth,
3. rupture of the WC/WC and WC/Co interphase,
4. deformation in the Co binder phase accompanied by fcc-hcp transformation.

That is why the accumulation of fracture damage during indentation loading results in an additional decline in *H*_0_ as compared to that due to the drop in GND density at the increase in indentation size. Therefore, one of the aims of the paper is to share the strain and fracture modes of the WC-Co composite ISE.

### 1.3. Nanoindentation

Detailed description of the influence of the microstructure on the ISE parameters of WC-Co cemented carbides is believed to provide an understanding of their mechanical behavior. Generally, WC-Co cemented carbides have a two-phase microstructure (WC and Co based binder). Therefore, examining the nanomechanical behavior of WC-Co composite phases will allow further understanding of the deformation and fracture mechanisms of these phases as well as the influence of the composition and technology route on their characteristics. Thus, the major objectives of composite nanohardness analysis are: (i) to study the nanoindentation behavior of SPSed WC-Co composites (Table 1), and (ii) to examine the SGP ISE model to explain the ISE of WC and Co phases of sintered WC-Co composites.

Duszová et al. [17] examined WC-Co cemented carbides using nanohardness measurements in the range of loads 0.1–10 mN. The mechanical properties of the separate WC grains with different crystallographic orientation were shown. The authors of [17] demonstrate that the hardness of the WC crystals and Co phase is in the ranges 20–60 GPa and 5–20 GPa, respectively. A relatively large variation of experimental data during indentation at the load of 1 mN was observed. The difference in WC grain nanohardness is found to depend on grain orientation relative to the normal force direction, and the average hardness values are the following: H_basal_ = 40.4 GPa and H_prismatic_ = 32.8 GPa at the indentation load of 10 mN. The authors of [17,18] note that the main reason for the variation in hardness at low indentation loads is the presence of damaged areas and residual stresses in the WC-Co composite. In the case of higher loads, the deformation and fracture processes in the “mix-phase” area below the indenter will result in specific features of mechanical behavior during nanoindentation and indentation. Nonetheless, there is a lack of studies of cemented carbides with nanoindentation at higher loads. Hence, the study of the nanomechanical properties of WC-Co cemented carbides at relatively high applied loads and examination of the ISE in these conditions is one of the aims of this paper.

### 1.4. Influence of WC-Co Cemented Carbide Alloying

The presence of the tough Co binder phase [19] in the WC-Co microstructure is shown to be of great importance, and the Co phase properties depend on its alloying during the sintering process. As shown in [20], only a small amount of carbon and some metals may be dissolved in the Co matrix at ambient temperature. These atoms are located at the damaged grain boundaries, which leads to increased grain growth. This process needs to be inhibited by advanced alloying (doping with Cr_3_C_2_, VC and other carbides) or heating methods such as spark plasma sintering (SPS), which result in the formation of mixed carbide phases, reducing grain boundary migration at sintering temperatures and improving corrosion resistance [20]. Most of the studies in the literature are focused on examining grain growth during or after liquid phase sintering [21,22,23]. The influence of VC and Cr_3_C_2_ additions on the WC grain growth features is examined the mentioned works. For example, TaC and VC form complex carbides such as (Ta,W,V)C_x_ which inhibit grain growth, whilst Cr_3_C_2_ dissolves in the Co binder phase that inhibits grain growth [23,24]. The authors of papers [21,22] describe the metallurgical features of WC-Co cemented carbide microstructure formation and made detailed characterization of their mechanical properties such as hardness and fracture toughness. Nevertheless, there is a lack of detailed investigations of the deformation behavior of these WC-Co based cemented carbides and, in particular, SPSed WC-Co based composites.

Hence, the aims of the paper are the following: (i) to examine the microhardness and nanohardness behavior of SPSed WC-Co based composites doped with Cr_3_C_2_, TaC-NbC, TiC and VC; (ii) to define some parameters characterizing the microstructure–microhardness relationship; and (iii) to characterize the nanomechanical properties of WC-Co cemented carbide phases in order to separate the deformation and fracture processes during the indentation process.

## 2. Materials and Methods

WC-6Co nanocomposite powder (purity: 99.9%, APS: 40–80 nm), Cr_3_C_2_ powder (purity: 99.9%, APS: 6 µm), TaC-NbC powder (purity: 99.9%, APS: 3 µm, ratio: 60:40) delivered by Inframat Advanced Materials, Manchester, USA and VC powder (purity: 99.9%, APS: 600–800 µm), TiC powder (purity: 99.8%, APS: 50 nm) delivered by Kamb Import-Export, Warsaw, Poland were used as the initial powders.

The SPS procedure was similar to that described by the author in reference [24]. The WC-6Co, WC-6Co-*x*Cr_3_C_2_, WC-6Co-*x*TaC-NbC, WC-6Co-*x*TiC, and WC-6Co-*x*VC (*x* = 0.5 and 1.0 wt%) powder mixtures were made by mechanical mixing using a Turbula T2F (WAB, Muttenz, Switzerland) shaker-mixer and then densified by SPS employing an HP D 25/3 (FCT Systeme, Rauenstein, Germany) furnace. The compaction pressure was kept constant at 80 MPa throughout the sintering process. The sintering temperature of 1200 °C was reached at the heating rate of 400 °C/min. The dwell time of 5 min was applied, after which the SPSed compacts were cooled to ambient temperature. The sintering chamber vacuum was set at 0.05 mbar for all the stages of the SPS processes. Samples with a diameter of 20 mm and thickness of approx. 3.2 mm were produced.

Microscopic observations were performed by scanning electron microscopy (SEM) by means of a MIRA 3 (TESCAN, Brno, Czech Republic) microscope.

The studied materials were four groups of WC-Co cemented carbides with additions and a benchmark WC-6Co (Table 1). Therefore, eight grades corresponding to different combinations of WC-Co and Cr_3_C_2_, TaC-NbC, TiC, and VC carbides were studied. The key microstructural parameters along with the composition of the examined materials are shown in Table 1. The mean WC grain size (*d_wc_*) was determined by image analysis using SEM backscattered micrographs taken from polished surfaces. The Co binder content values were constant. The values for the Co binder thickness (*λ_Co_*) and contiguity (*C_WC_*) were evaluated based on experimental data [7]. Contiguity *C_WC_* is defined as the ratio of grain boundary area of WC crystals and total grain boundary area:

(4) $$C_{WC}=\frac{2N_{WC/WC}}{2N_{WC/WC}+N_{WC/Co}},$$

where *N_WC/WC_* and *N_WC/Co_* are the numbers of WC/WC and WC/Co interfaces that are intercepted, respectively. Co binder thickness (*λ_Co_*) is defined by the empirical relationship:

(5) $$\lambda_{Co}=\frac{2f_{Co}}{N_{WC/Co}},$$

where *f_Co_* is the volume content of the Co binder phase. All the measurements were done by the line intercept method on suitable micrographs using ImageJ (Laboratory for Optical and Computational Instrumentation, University of Wisconsin, Madison, WI, USA) software.

Hardness measurements were carried out by applying loads of 4.905 and 19.62 N for 15 s using an FM-800 (Future-Tech, Kawasaki, Japan) hardness tester in accordance with the PN-EN 23878 standard. The Vickers indentation nanohardness measurements were made with a Picodentor HM500 (Fisher, Sindelfingen, Germany) nanoindenter (ISO 14577-1 standard [25]). The initial applied load was 50 mN and the dwell time was 5 s. The indentation of WC-6Co with the load of 50 mN results in an approximate maximum indent depth of 150–170 nm as compared to that of 20–60 nm for indents obtained under the load of 1 mN [17]. Hence, indentation with a depth of 150–170 nm results in deformation by a Vickers indenter of the surface area of approximately 0.8 µm^2^, which is in the range of a 1 µm WC grain size. Using multiple loading–unloading steps up to 500 mN will allow the deformation area to be considerably raised (up to 70 µm^2^), which includes approximately 30–50 WC grains of a 1 µm size. Thus, multiple indentation tests with loads in the range of 50–500 mN will allow the ISE of WC-Co based cemented carbides to be estimated in the various loading regimes at the submicro-scale.

The fatigue behavior of WC-Co cemented carbides was examined in the bending and compression modes [20,23]. Additionally, indentation tests [24] were used to characterize the fatigue and hardness. WC-Co cemented carbide indentation fatigue behavior characterization was made by Duszová et al. [8] with a Berkovich indenter in the load range of 25–200 mN. The dislocation structure and crack formation were examined using FIB/TEM of the areas under the indenter. The authors of [7] note that the microstructure damage process proceeds in the first cycles of indentation loading. However, detailed examination of the loading regimes on the microstructure damage processes and their relation to ISE phenomenology was not carried out.

**Figure 1:**
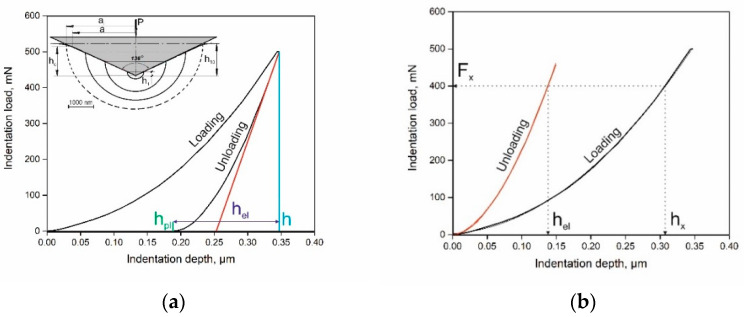
(**a**) Nanohardness diagram and (**b**) plastic deformation parameter calculation schematics; 10-cycle nanoindentation scheme is shown in insert of Figure 1a.

Before the nanoindentation tests, the grinded samples were processed with 0.5 μm diamond paste. The samples were indented with the load of 50 mN, then unloaded and repeatedly re-loaded to 10 cycles with loads of 50, 100, 150, 200, 250, 300, 350, 400, 450 mN (Figure 1a, insert) The following nanohardness measurement at the maximal load of 500 mN was performed after 10-cycle loading with a step of 50 mN and loading rate of 10 mN/s.

In accordance with the authors of [18], the nanoindentation route is shown in Figure 1 as a diagram “indentation load–indentation depth” (Figure 1a). In the present work, a Vickers tip was used. Loading increased along the “loading” line until the indentation load became maximal. This resulted in a maximum depth of penetration shown as *h*, which was identified by the perpendicular drawn on the depth indentation depth axis. The unloading curve showed the final depth, which was elastically recovered with *h_el_* (Figure 1a). Calculation of the plastic deformation depth, *h_pl_* = *h_x_* − *h_el_*, was performed based on determining the square root of *h_el_* of the polynomial approximation equation of the unloading curve (Figure 1b). The multiple loading–unloading indentation schematics are shown in the insert of Figure 1a.

## 3. Results

### 3.1. Microstructure and Hardness

The microstructure parameter determination results shown in Table 1 demonstrate that the Co volume fraction, *f_Co_*, is in the range of 10%, whilst the WC grain size (*d_WC_*) varies within 200–400 nm due to the influence of grain growth inhibitor elements on the microstructure formation during sintering [24]. It should be noted that the WC grain size in the SPSed composites is smaller than that of WC cemented carbides compacted at 150 MPa and sintered at the temperature of 1400 °C under vacuum for 60 min [26] (*d_WC_* = 1.9–5.3 µm). The Co binder mean free pass, *λ_Co_*, is sensitive to alloying. It varies in the range of 1.25–5.06 nm in spite of the fact that the Co volume fraction does not change considerably. The carbide contiguity (*C_WC_*) does not vary to a great extent (0.69–0.81). The effect of Cr_3_C_2_ alloying is seen.

**Figure 2:**
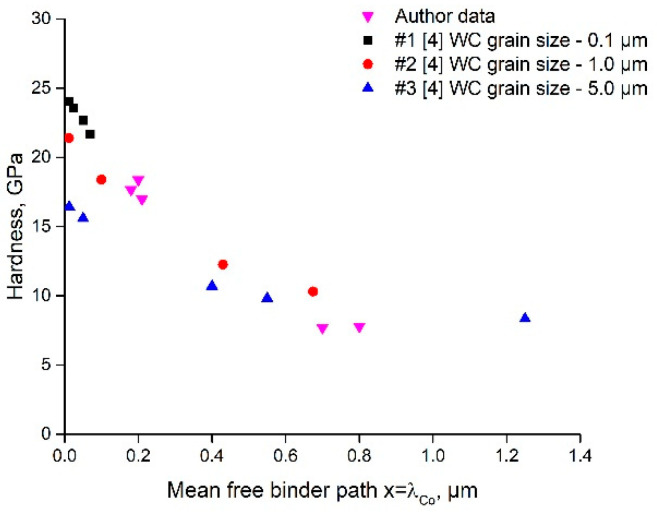
Theoretically calculated curves (Engqvist et al. [4] model, Equation (1)) “hardness vs. mean free binder path *λ_Co_*” for three WC grain sizes (0.1, 1.0, and 5.0 µm) and experimental data (Table 1).

Figure 2 presents an *H_CC_* = *f*(*λ_Co_*) graph for three *d_WC_* and experimental data obtained in the work. It can be concluded from Equation (1) that the experimental data of WC-6Co-0.5TiC and WC-6Co-1.0TiC (grades #6, 7) fit well the area between the curves of WC grain sizes of 0.1 and 1.0 µm, whilst the data of grades #1, 2, 3 fit the curve of the WC grain size of 0.1 µm.

The Equation (1) model of the WC-Co composite hardness as a function of WC grain size and Co mean free pass is based on physical understanding of the deformation processes, expressed in the following assumptions and statements.

- In the case of thin Co binder layers between the WC grains, the layers have a deformation resistance similar to that of the neighboring WC grains.
- Equivalent flow stress *σ_CC_* = *H_CC_*/3 falls exponentially (≡*e*^−*λCo*/*k*^) towards the properties of bulk Co with an increasing thickness of the Co binder layer.
- The Hall–Petch relation to calculate the hardness of polycrystalline WC is proven to give good estimates of the hardness for grain sizes from 0.25 to 5 μm.

**Figure 3:**
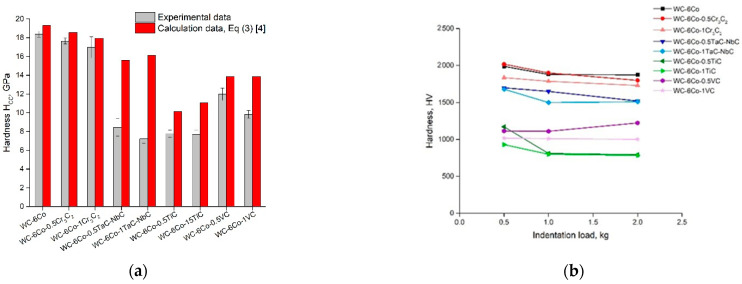
(**a**) Comparison of experimental (indentation load of 19.6 N) and calculated hardness of WC-Co cemented carbides; (**b**) indentation size effect on Vickers hardness measurements (designation of powder compositions is shown in Table 1).

A comparison of the experimental and calculated hardness of WC-Co cemented carbides is shown in Figure 3a. The experimental results reveal that the hardness numbers determined based on Equation (1) are 25% in agreement with the measurement results for various compositions and the hardness range of 800–2000 MPa. Nonetheless, it can be noted that the calculated values of *H_CC_* for all the studied cemented carbide compositions are higher than those obtained during hardness testing (Figure 3a). The real drop in hardness is about 12–28% of the calculated *H_CC_*. A possible reason for such mechanical behavior of WC-Co cemented carbides seems to be the nucleation, propagation and accumulation of cracks that lead to diminishing WC-Co composite flow stress or hardness. For this reason, the ratio of experimental and calculated values of microhardness may be an approximate indication of WC-Co sensitivity to hard metal damage processes during indentation. More detailed examination of this criterion based on microstructure analysis of areas under the indenter will be made in future work.

### 3.2. Microhardness Indentation Size Effect Evaluation

The results (Figure 3b) demonstrate the small hardness ISE which is in the range of the Δ*H_CC_/H_CC_* parameter. Nevertheless, it seems to be reasonable to define one of the main ISE parameters—the intrinsic hardness, *H*_0_, based on Equation (2):

(6) $$H^{2}=H_{0}^{2}h^{*}\left(\frac{1}{h}\right)+H_{0}^{2},$$

**Figure 4:**
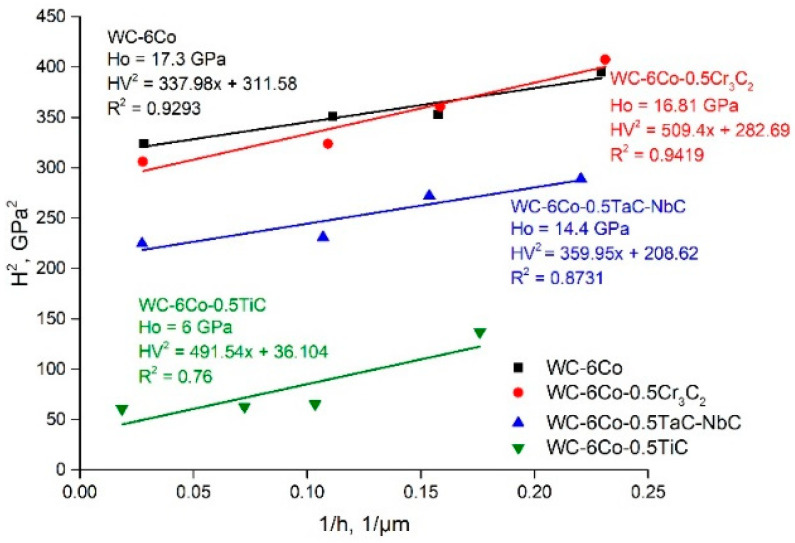
Hardness indentation size effect (ISE) of WC-Co cemented carbides at various indentation loads.

The results of *H*_0_ evaluation based on linear approximation of experimental functions *H_cc_*^2^ = *f*(1/*h*) are shown in Figure 4. It is clearly seen that the simulation veracity of linear approximations of the experimental data is in the range of R^2^ *=* 0.76–0.94, which indicates the conformity of SGP theory to the experimental results. *H*_0_ is in the range of 6.0–17.3 GPa for various WC-Co based cemented carbides. The reason for such a difference between intrinsic hardness *H*_0_ and real *H_CC_* is believed to be the influence of microstructure damage effects on the mechanical properties of cemented carbides.

Whilst the calculation of *H_CC_* is based on considering the dislocation generation and movement processes that result in hardening effects, it seems to be reasonable to assume that crack nucleation, propagation and accumulation lead to dislocation release, lowering the dislocation density, and result in strength degradation. The hardness ISE of WC-6Co based cemented carbides of the various types shown in Figure 4 demonstrates the conformity of SGP theory to the experimental results with a simulation veracity of 0.76–0.94. These results are in agreement with the Vickers functions *H*^2^ = *f*(1/*h*) of WC-6Co hard metals reported by Nabarro et al. [27].

Thus, the mechanical behavior of WC-Co composites during indentation is believed to be controlled by the deformation and fracture processes of WC carbides and Co binder, which needs to be analyzed at the submicro- and nanoscale based on the nanoindentation results presented in the paragraph below.

It can be noted that all the WC-Co carbide compositions may be divided into two groups: (i) high hardness WC-Co composites doped with Cr_3_C_2_ and TaC-NbC (HV > 1800 MPa), and (ii) low hardness WC-Co composites doped with TiC and VC (HV < 1300 MPa). It is interesting to mention that certain WC-Co cemented carbides in the second group (for example, WC-6Co-0.5VC) do not exhibit the ISE. The possible reason for such behavior may be modification of the Co binder phase due to alloying, and this effect will be discussed later.

### 3.3. Nanoindentation

**Figure 5:**
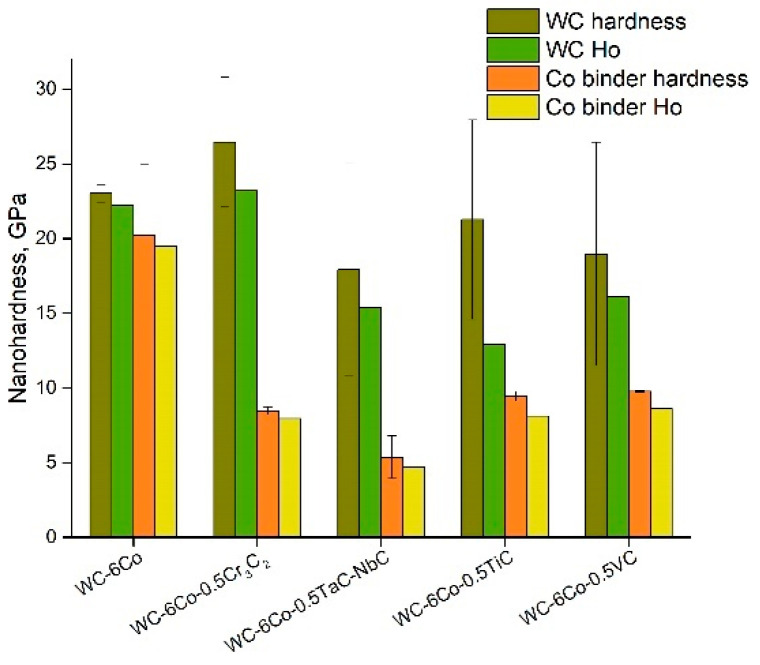
Nanohardness and Nix and Gao model parameters of WC-6Co based cemented carbides.

A comparison of the WC phase nanohardness of various WC-Co composites (Figure 5) demonstrates that only Cr_3_C_2_ doping leads to an effective increase in WC hardness due to the inhibition of WC grain growth as shown in papers [20,28,29]. The average WC grain size was not was not changed with the Cr_3_C_2_ additions during conventional liquid phase sintering [20]. However, some substantially larger WC grains were observed when the Cr_3_C_2_ addition was not used. Similar results were reported in [24]. It was found in [20] that the Cr_3_C_2_ addition influences the Cr/Co ratio, which results in a decline in the toughness of the composites owing to replacement of the Co phase with Cr-based carbides. Materials sintered by conventional sintering with VC additions [28] show an average WC grain size of less than 400 nm. SPSed materials possess a fine-grained microstructure, which is formed due to SPS, even without alloying elements. It should be noted that their addition associated with SPS results in obtaining submicro-scale cemented carbides of a 0.30–0.75 μm grain size (Table 1). The samples sintered by conventional sintering showed a low contiguity of WC grains (Table 1), which indicates a relatively homogeneous distribution of the Co phase similar to that in [28].

**Figure 6:**
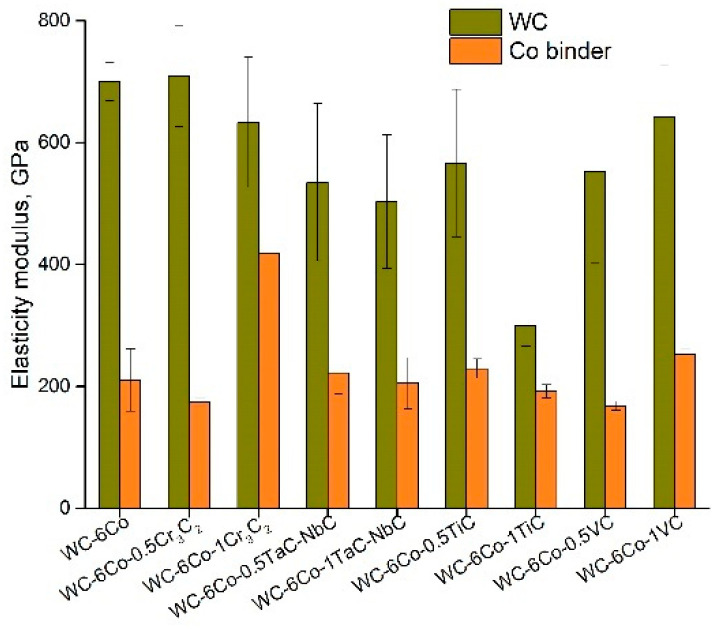
Elasticity modulus of WC-Co cemented carbide constituents.

**Figure 7:**
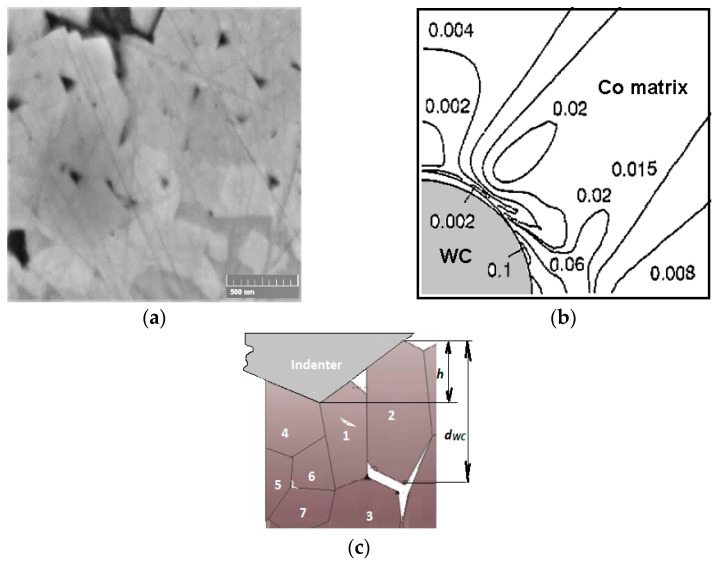
(**a**) Microstructure of WC-6Co composite; (**b**) von Mises-Fisher matrix strain distributions for σ_max_/σ_0_ = 1.72 and ε_2_ = 0.0244 [29] (courtesy of IOP Publishing Ltd.); (**c**) possible failure mechanisms and composite morphology during nanoindentation (*h_max_* = 250 nm at 50 mN for WC-6Co cemented carbide with *d_wc_* = 600 nm, Table 1).

It can be noted that doping the WC-Co composite leads to a decrease in the Co binder phase nanohardness for all the studied compositions (Figure 5), which reveals relative softening of the binder phase. Two possible reasons for such behavior may be assumed: (i) the dissolved alloying atoms make (at a lower shear stress) the processes of dislocation generation and movement easier, and (ii) the dissolved alloying atoms intensify pore and crack formation in the Co phase, which leads to a fall in the Co binder deformation resistance. Whilst the WC and Co phase deformation effects may be characterized by ISE parameter determination, cases of microstructure element fracture may be imagined by comparing the elasticity modulus of the constituent phase (Figure 6). The data reveal that the WC and Co phase moduli differ two or threefold, which results in high strain localization at the WC-Co phase interface. Moreover, the Co binder hardness is approximately 1.2–2.0 times lower than that of the WC carbide. Hence, the Co binder plastic deformation and localization are believed to occur at the WC-Co phase interface, which results in void nucleation. Void nucleation by inclusion debonding was FEM modelled by Xu and Needleman [30] based on equations and edge conditions individually specified for each phase and grain boundary. In the case of WC-Co cemented carbides, strain localization may be seen at the WC crystal–Co binder interface (see Figure 7b). Numerical simulation data indicate the great probability of void nucleation due to large strains in the Co matrix. The modelling data in [30] (Figure 7b) clearly demonstrate that for the plane strain case, the average plane strain of ε_2_ = 0.0244 results in the presence of some areas with ε_i_ = 0.1 at the interface, whilst the equivalent strain at the yield stress of the Co matrix is approximately 0.017. This means that the calculated value of ε_i_ = 0.1 is about five times higher than the source of Co matrix plasticity. The data (Figure 6) demonstrate that alloying greatly influences the elasticity modulus ratio, *E_WC_/E_Co_,* which is minimal for the WC-6Co-1Cr_3_C_2_ and WC-6Co-1TiC composites.

### 3.4. Nanoindentation Size Effect Evaluation

**Figure 8:**
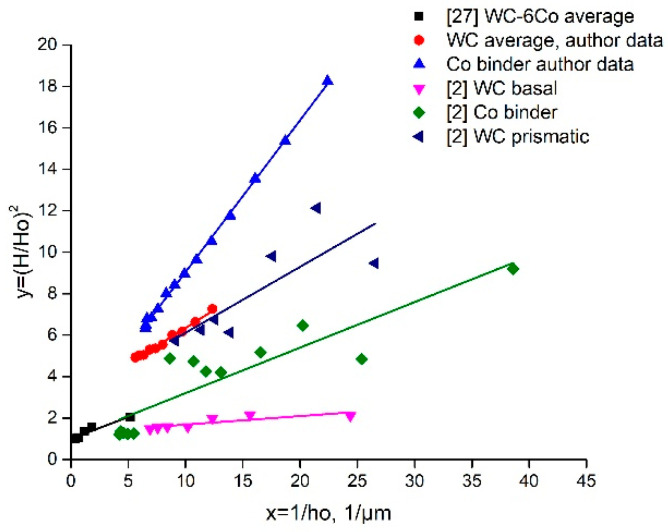
(*H*/*H*_0_)^2^ = *f*(1/*h*) dependences of WC-6Co composite constituents obtained by author and compared with data from works [2,27].

As shown in the introduction, the mechanisms of the ISE are well described on the basis of SGP theory. From this viewpoint, the nanoindentation mechanical behavior of WC-Co composites is believed to be evaluated by detailed characterization of the ISE parameters. ISE mechanisms may be defined based on separate examination of the nanohardness of the WC and Co binder grains. (*H*/*H*_0_)^2^ = *f*(1/*h*) dependences of the obtained WC-6Co composite constituents and shown by the authors of works [2,27] are presented in Figure 8. It is seen that the data in [27] coincide with the Co binder in paper [2]. Similar results are obtained in this work (WC grains) and paper [2] (WC prismatic crystals). Some difference in the h* parameter for the Co binder phase is seen, which may be attributed to the influence of the WC-Co cemented carbide composition. More detailed examination of the reasons for such WC-Co behavior is planned for future work.

The main SGP parameters characterize the formation of a stochastically stored and geometrically necessary dislocation network [14,15]. Based on Equation (2), where *H*_0_ is the hardness that would arise from the statistically stored dislocations only, or based on Equation (3) in the absence of any geometrically necessary dislocations, parameter h* characterizes the strain gradient sensitivity of the hardness [27]:

(7) $$h^{*}=\frac{81}{2}b\alpha^{2}tg^{2}\theta {\left(\frac{\mu }{H_{0}}\right)}^{2},$$

where *α* is taken to be ½, *b* is the Burgers vector, *μ* is shear modulus, and *Θ* is a half-angle of the indenter pyramid. Equation (2) predicts the linear dependence (*H*/*H*_0_)^2^ = *f*(1/*h*), and *H*_0_ may be determined from the equation:

(8) $$H^{2}=H_{0}^{2}+\frac{81}{2}b\alpha^{2}\mu^{2}tg^{2}\theta \frac{1}{h},$$

**Figure 9:**
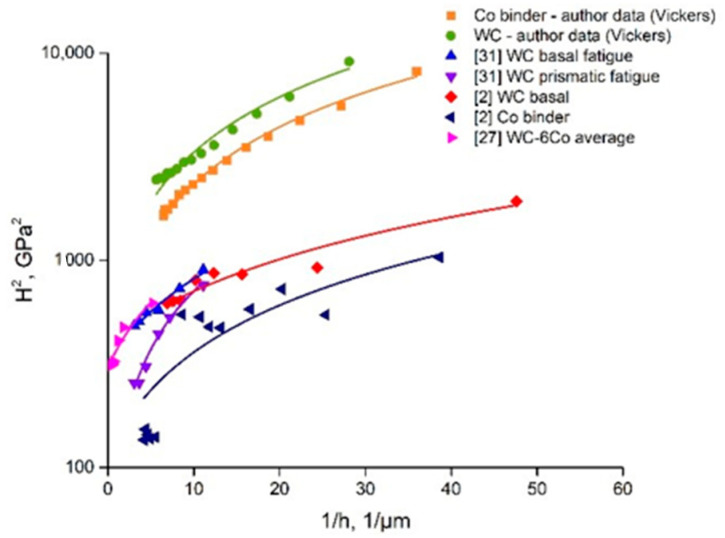
Comparison of linear approximations of *H*^2^ = *f*(1/*h*) functions of WC-6Co cemented carbide nanoindentation curves (curve designations are on the graph).

It must be noted that h* is a function of many parameters (Equation (7)), and depends on the SSD density described by *H*_0_, whilst 81*α*^2^/3 is a numerical factor, and *bμ*^2^ is a material constant [27]. For this reason, accurate determination of intrinsic hardness *H*_0_ based on the linear approximation of function Equation (8) is important. Examples of the linear approximations of the *H*^2^ = *f*(1/*h*) functions calculated on the basis of the author’s nanohardness measurement results and data from works [2,27,31] are shown in Figure 9.

First of all, one can note that the results of works [2,27,31] are based on Berkovich nanoindentation measurements contrary to Vickers measurements in our case. As shown in [32], the difference between the Vickers and Berkovich nanoindentation results depends both on the material and the indenter type. The authors of [32] observed distinctly different mechanical behaviors for composite materials. Therefore, the obtained difference in the *H*^2^ = *f*(1/*h*) curves for Vickers and Berkovich nanoindentation looks reasonable because the pyramid sample surface contact area of the Vickers tip is larger than that of the Berkovich tip by approximately 30% at constant projected areas for both indenter types. The Berkovich projected area is $A_{proj}=3\sqrt{3}h^{2}tan^{2}65.3^{{}^{\circ}}=24.56h^{2}$, whilst the Vickers projected area is $A_{proj}=4h^{2}tan^{2}68^{{}^{\circ}}=24.504h^{2}$. Thus, the hardness values defined by Vickers nanoindentation are higher than those of the Berkovich ones. Such behavior is clearly seen in Figure 9. These results are in accordance with work [32]. The described influence of indenter geometry weakens at large indentation depths. The possible reasons for such behavior seem to be more uniform deformation of the metal underneath the indenter, and the microstructure damage processes are similar to those shown by the diagram presented in Figure 7c. The possible areas of crack initiation may be at the grain boundaries and grain core.

Figure 9 demonstrates that the literature data of nanoindentation with a Berkovich tip are similar to those obtained in our experiments with a Vickers tip. The simulation veracity of the linear approximations is in the range of R^2^ = 0.80–0.95, which reveals the trustworthiness of the Nix and Gao model (Equation (2)). The comparison of intrinsic hardness *H*_0_ with the nanohardness of the examined WC-Co composites at the indentation load of 50 mN (Figure 5) demonstrates that the deformation process of each phase (both WC and Co binder) has its own specific features, which depend on the material composition. Hence, it seems to be interesting to evaluate the density of statistically stored dislocations, *ρ_S_*. Based on Equation (3), $H_{o}=3\sqrt{3}\alpha \mu b\sqrt{\left(\rho_{S}\right)}$, one can note that *ρ_S_* is proportional to *H*_0_^2^:

(9) $$\rho_{s}=\frac{H_{0}^{2}}{27{\left(\alpha \mu b\right)}^{2}},$$

**Figure 10:**
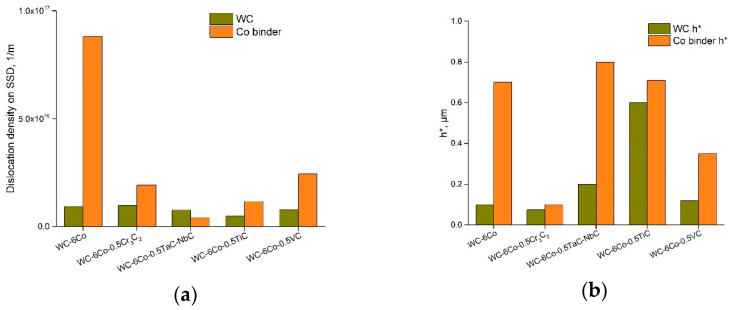
(**a**) SSD density of WC-Co cemented carbides determined based on Equation (9); (**b**) intrinsic length (h*) parameter of WC-Co composite microstructure constituents.

The calculation results for each group of examined WC-Co cemented carbides are presented as a diagram in Figure 10a.

It is seen that the various carbide doping of the WC-6Co composite do not considerably influence the WC dislocation density. On the contrary, the Co binder phase is quite sensitive to carbide alloying. If the basic WC-6Co composite exhibits *ρ_S_* ≈ 8.8 × 10^16^ m^−1^, *ρ_S_* ≈ 1.9 × 10^16^ m^−1^ is defined for the WC-6Co-0.5Cr_3_C_2_ composition. The calculation results are in agreement with work [27], and it looks like alloying diminishes the hardening effect of the Co phase during nanoindentation by 3–4 times. The possible reasons for such an effect will be examined in future.

**Figure 11:**
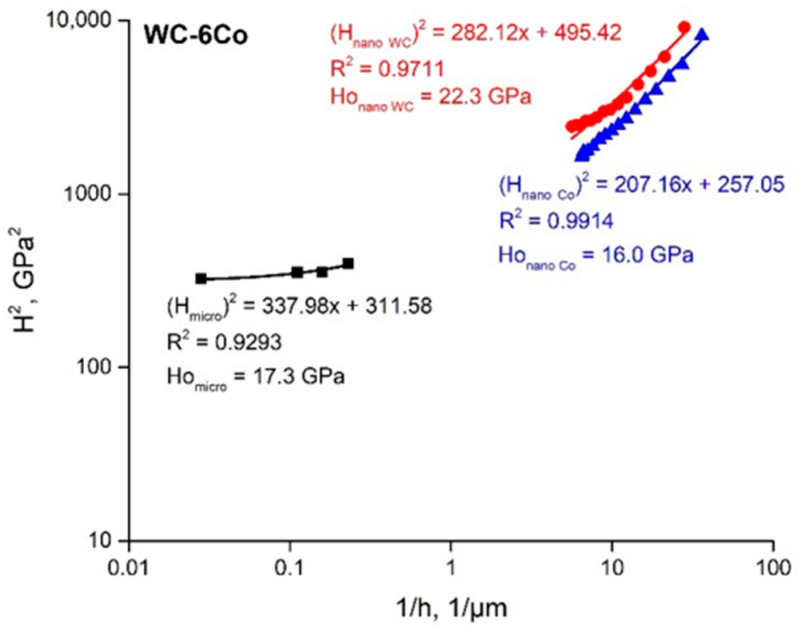
Comparison of *H*_0 *nano*_ of WC and Co binder and *H*_0 *micro*_ of WC-6Co cemented carbide (Figure 4).

WC-Co based cemented carbide fracture processes may be characterized by comparing the intrinsic hardness values defined by nano- and microindentation tests because it is well known that hardness tests result in crack generation and propagation. Comparison of the *H*_0 *nano*_ of the WC carbide and the *H*_0 *micro*_ of the WC-6Co cemented carbide (Figure 11) demonstrates the difference Δ*H*_0_ ≈ 5 GPa. The *H*_0 *nano*_ of the Co binder is slightly less than that of the WC-6Co cemented carbide. *H*_0 *micro*_ is characteristic of material mechanical behavior in an indent area of about 200 × 200 µm^2^, which means that this parameter reveals the average *H*_0_ value of both phases available in the indent area. The value of Δ*H*_0_ seems to depend on the accumulation of fracture defects both in the WC and Co binder phases during indentation with a large imprint depth. Taking into account the fact that the linear approximation of the Nix and Gao function (Equation (6)) is based on the strain gradient effect and *H*_0_ is defined by the density of SSDs, it can be assumed that the drop in Δ*H*_0_ may be a result of composite softening due to both the release of dislocations in the crack sites and an increase in the fracture defect density. Nevertheless, it is difficult to divide these effects based on simple comparison of the *H*_0_ parameters because the conditions of hardness and nanohardness tests are quite different. From this viewpoint, it seems to be reasonable to use only nanoindentation methodology to separate the deformation and fracture modes of WC-Co based cemented carbide mechanical behavior.

As shown above, the Nix and Gao deformation behavior of the WC-Co cemented carbide phases is described by the intrinsic length h* parameter, which is characterized by a decrease in hardness with indentation depth due to the diminishing GND density. The obtained h* calculation data (Figure 10b) demonstrate that the WC grains show low sensitivity of GND density to the indentation depth, whilst the Co binder phase is more sensitive to this effect. Such behavior may result from the higher strains of the Co binder achieved at a large indentation depth and the possible localization of strains at the WC-Co binder interface (Figure 7b). Additionally, some voids as well as crack nucleation and propagation during indentation may enhance this effect. Moreover, as shown above, the decrease in hardness with indentation depth seems to be the result of softening due to fracture processes in the case of WC-Co cemented carbides. Based on this, it seems to be reasonable to use the coefficient of decrease in hardness with indentation depth in the function *H*^2^ = *f*(1/*h*), which usually fits linear approximation very well (see Figure 9).

### 3.5. ISE at Multicycle Indentation Loading

To define the effect of damage of the WC-Co composite microstructure on the Nix and Gao model parameters, 10-cycle nanohardness tests were carried out (Figure 1a, insert). Using the loading-unloading regime allows the fracture processes to be enhanced due to the difference in the elastic modules of the WC and Co binder phases. The described 10-cycle loading regime is severe and is believed to result in intensive crack generation. The experimental data of nanohardness measurement after usual and fatigue loading [2,27,31] are shown in Figure 8, together with the author’s data. Additionally, these data are compared with the basic SGP parameters calculated from the nanohardness measurements of the WC-6Co based composites after sintering (Figure 9). It must to be noted that multicycle indentation loading leads to the nucleation and accumulation of microstructure damage defects as was clearly shown by Duszová et al. [8,31]. Moreover, the authors of [31] state that the damage events of WC-6Co based composites occur during the first 10 cycles of indentation loading. These damage processes result in a change in the SGP parameters of the WC-6Co carbide indentation curve. As shown in a previous discussion, the main characteristic of the WC-6Co cemented carbide mechanical behavior is intrinsic hardness *H*_0_. The data in [31] after 100 cycle loading are the following: the WC prismatic crystals exhibit *H*_0_ = 20.1 GPa, and 100 cycle loading results in *H*_0_ = 5.6 GPa for these crystals. The Co binder phase exhibits identical behavior: *H*_0_ =10.6 GPa, and 100 cycle loading results in *H*_0_ = 4.8 GPa. The main possible reason for the considerable fall in *H*_0_ is the association of two microstructure formation processes during nanoindentation: i) GND density decrease, and ii) damage defect accumulation, which results in a release of SSDs.

**Figure 12:**
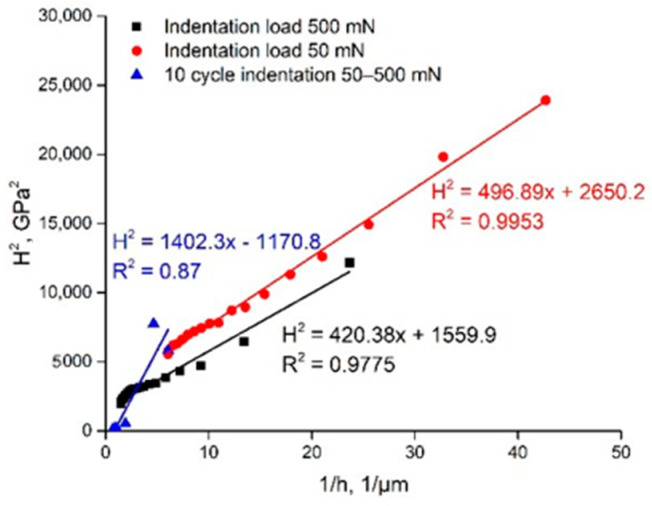
Comparison of linear approximations of *H*^2^ = *f*(1/*h*) functions of WC in WC-6Co cemented carbide nanoindentation curves (curve designations are on the graph).

The 10-cycle nanohardness measurement results of the WC phase in the WC-6Co cemented carbide are shown in Figure 12 as the dependences *H*^2^ = *f*(1/*h*) approximated by linear functions. Comparison of the indentation curves of the WC phase (average of basal and prismatic WC grain orientations) for loads of 50 and 500 mN demonstrates similar Nix and Gao linear function parameters (Figure 12). Nonetheless, the 10-cycle indentation curve constructed based on 50–500 mN cycles exhibits considerably different behavior with negative values of *H*_0_. The coefficient of decrease in hardness for 10-cycle indentation loading is about 1402.3 GPa·µm as compared to 420–500 GPa·µm for usual continuous indentation.

**Figure 13:**
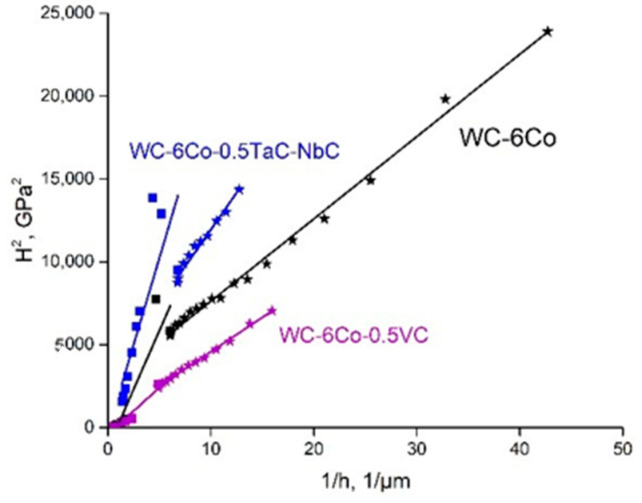
Comparison of linear approximations of *H*^2^ = *f*(1/*h*) functions of WC in WC-6Co based cemented carbide nanoindentation curves (curve designations are on the graph).

Such an effect of growth of the coefficient of decrease in hardness seems to be the result of the generation and accumulation of fracture defects due to the loading–unloading indentation regime. Similar results are obtained for two other WC-Co based compositions (Figure 13). The difference in the coefficient of decrease in hardness for 10-cycle indentation loading is about 3–4 times, providing negative values of *H*_0_. Thus, a clear indication of the fracture processes during indentation was found.

**Figure 14:**
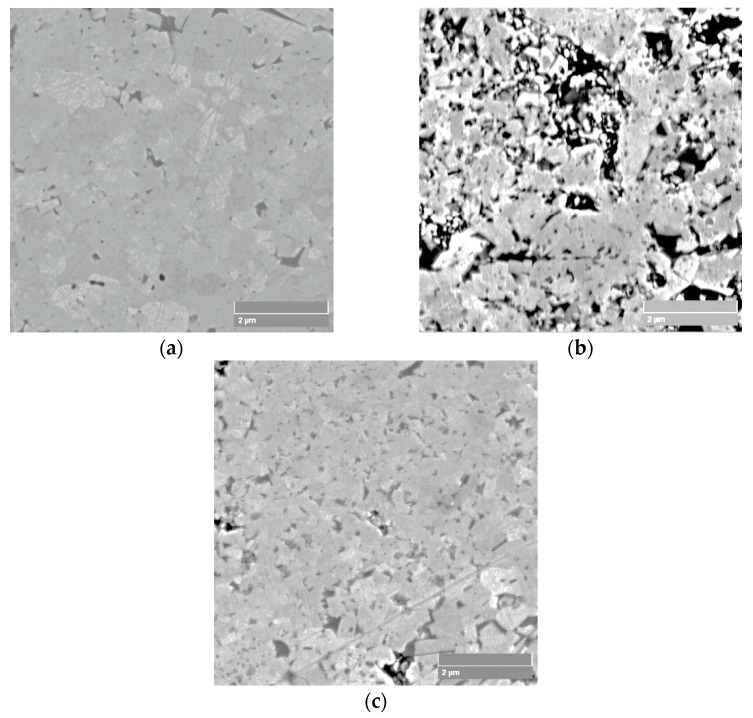
SEM micrographs of microstructure of: (**a**) WC-6Co, (**b**) WC-6Co-0.5TaC-NbC, and (**c**) WC-6Co-0.5VC cemented carbides.

It can be noted that WC-6Co-0.5TaC-NbC exhibits the highest sensitivity of the coefficient of decrease in hardness, perhaps because of the higher content of Co binder phase (Figure 14b) as compared to WC-6Co (Figure 14a) and WC-6Co-0.5VC (Figure 14c). A more detailed physical interpretation of these observations is still to be made.

## 4. Conclusions

The main results of the current work are the following:

- The microstructure parameters and their influence on the hardness of cemented carbides are defined. The WC grain size is controlled by cemented carbide alloying with Cr_3_C_2_, TaC-NbC, TiC, and VC.
- The calculated microhardness of WC-Co cemented carbides for all the studied compositions is higher than that obtained during hardness testing, which reveals the possible development of fracture processes during the micro-indentation process and declining WC-Co composite flow stress. Therefore, the ratio of experimental and calculated values of microhardness is shown to be an approximate indication of WC-Co sensitivity to hardmetal damage processes during indentation.
- The mechanical behavior of WC-Co cemented carbides during micro- and nanoindentation indicates the deformation and fracture mechanisms of the WC and Co-based phases. It was found that both processes influence the parameters of the strain gradient plasticity (Nix and Gao) functions.
- Nix and Gao constants are determined for the WC and Co phases of the studied cemented carbide grades. It was shown that the obtained results coincide with literature data for the Co binder phase as well as for WC prismatic and basal crystals. Some difference in the h* parameter for the Co binder phase may be attributed to the influence of the WC-Co cemented carbide composition.
- WC-Co based cemented carbide fracture processes may be characterized by comparing the intrinsic hardness values defined by the nano- and microindentation tests. It was found that *H*_0 *nano*_ of the WC carbide and *H*_0 *micro*_ of the WC-6Co cemented carbide differ by Δ*H*_0_ ≈ 5 GPa, which reveals the possible occurrence of fracture processes at large indentation depths.
- Comparison of the indentation curves of the WC phase for loads of 50 and 500 mN demonstrates similar Nix and Gao linear function parameters. It was found that the 10-cycle indentation curve constructed based on 50–500 mN cycles exhibits anomalous behavior with negative values of *H*_0_.
- The difference in the coefficient of decrease in hardness for 10-cycle indentation loading is about 3–4 times, providing negative values of *H*_0_. Therefore, these features might be used as an indication of fracture processes during indentation.

## Figures and Tables

**Table 1:** WC-Co composite designation and microstructural data (Co volume fraction, *V_Co_*; WC grain size, *d_WC_*; Co mean free pass, *λ_Co_*, and carbide contiguity, *C_WC_*).

Group	Series Number	Composite Designation	Microstructure Parameters of SPSed WC-Co Cemented Carbides			
			*f_Co_*, vol%	*d_WC_*, nm	*C_WC_*, -	*λ_Co_*, nm
0	1	WC-6Co	10.12	416	0.81	5.06
1	2	WC-6Co-0.5Cr_3_C_2_	10.06	284	0.74	1.25
	3	WC-6Co-1Cr_3_C_2_	9.99	-	-	-
2	4	WC-6Co-0.5TaC-NbC	10.10	204	0.69	2.88
	5	WC-6Co-1TaC-NbC	10.07	-	-	-
3	6	WC-6Co-0.5TiC	10.02	226	0.75	2.22
	7	WC-6Co-1TiC	9.91	-	-	-
4	8	WC-6Co-0.5VC	10.04	244	0.78	3.98
	9	WC-6Co-1VC	9.96	-	-	-

## Data Availability

Data is contained within the article.
